# Combined quality of life and posttraumatic growth evaluation during follow-up care of patients suffering from oral squamous cell carcinoma

**DOI:** 10.3892/mco.2021.2351

**Published:** 2021-07-15

**Authors:** Georg Hoene, Rudolf M. Gruber, Johanna J. Leonhard, Bernhard Wiechens, Boris Schminke, Philipp Kauffmann, Henning Schliephake, Phillipp Brockmeyer

**Affiliations:** 1 Department of Oral and Maxillofacial Surgery, University Medical Center Goettingen, Goettingen D-37575, Germany; 2 Department of Orthodontics, University Medical Center Goettingen, Goettingen D-37575, Germany

**Keywords:** oral squamous cell carcinoma, oral squamous cell carcinoma, health-related quality of life, posttraumatic growth, tumor outcome

## Abstract

Oral cancer therapy is associated with a loss in health-related quality of life (HRQOL) and can also lead to post-traumatic growth (PTG). The current study analyzed the relationship between HRQOL, PTG and influencing clinical factors after treatment. The coherent clinical data of 15 patients were retrospectively analyzed over a 1-year study period. HRQOL and PTG were studied using the University of Washington Quality of Life Version 4 (UW-QOL v4) and Posttraumatic Growth Inventory (PTGI) questionnaires. The results revealed that HRQOL was significantly decreased in a pre- to postoperative manner (P=0.011). Sex demonstrated a nearly significant effect on HRQOL (P=0.058). PTG was experienced the most after surgery, and continuously decreased over the 1-year study period. Patient age had a significant effect on PTG (P=0.040). A significant correlation was also established between HRQOL and PTG (P<0.05). HRQOL and PTG are important influencing factors during postoperative tumor follow-up care and should be simultaneously recorded to address individual patient needs and improve quality of treatment.

## Introduction

Tumors of the head and neck region are a heterogeneous group of neoplasms and are among the most common cancer worldwide (1). With a proportion of 90%, squamous cell carcinoma (SCC) is the most common histological type (2,3). The main risk factors for the development of oral squamous cell carcinoma (OSCC) are chronic alcohol and/or nicotine abuse with synergistic potentiation (4). Surgical therapy for extensive OSCC is associated with a functional loss in stomatology, aesthetic restrictions, and a deterioration in a patient's health-related quality of life (HRQOL). HRQOL evaluation is a multidimensional concept comprising positive and negative components (5-7). Besides physical, psychological, and social dimensions, marital status and spiritual well-being are also important influencing factors (8,9). Various questionnaires have been developed to assess HRQOL in different dimensions (10-13). Among them, the University of Washington Quality of Life Questionnaire version 4 (UW-QoL V4) is a widely used tool for HRQOL evaluation in patients with OSCC (14), and is routinely used in many centers worldwide during postoperative follow-up care.

Cancer diagnosis and treatment are traumatic life events that can trigger post-traumatic stress disorder (PTSD) (15,16), but can also lead to subjectively positive effects, such as post-traumatic growth (PTG) (17,18). PTG does not refer to the traumatic event itself, but the subjectively perceived positive change in the way in which the traumatic experience is dealt with (17). Many sociodemographic factors, such as age, sex, social support, religion, educational level, income, resilience, HRQOL, and disease severity, have been discussed to affect PTG. PTG can be assessed using the PTG-inventory (PTGI) questionnaire developed by R. Tedeschi and L. Calhoun (19).

Both HRQOL and PTG are important influencing factors during post-therapy cancer follow-up care. For this reason, a better understanding of the relationships between HRQOL, PTG, and influencing clinical factors is important in specifically addressing individual patient needs and improving quality of treatment. To the authors' knowledge, this study is the first to report a combined HRQOL and PTG evaluation in patients with OSCC.

## Materials and methods

### 

#### Patients and data acquisition

Coherent clinical data (seamless data acquisition over the entire observation period) of 15 patients with OSCC mainly treated surgically between January 2011 and June 2012 were retrospectively analyzed. Data included patient characteristics, such as age, sex, marital status, and denominational affiliation; pathological characteristics, such as localization of the primary tumor and TNM classification; and therapy characteristics, such as surgical reconstruction modality and adjuvant therapy. Clinical data of patients can be found in Table I. HRQOL and PTG were recorded using the UW-QoL V4 and PTGI questionnaires (14,19,20). UW-QoL V4 was completed one day preoperatively and during postoperative tumor follow-ups (after 1/2, 1, 3, 6, 9, and 12 months); and it consisted of 12 questions concerning the domains of pain, appearance, activity, recreation, swallowing, chewing, speech, shoulder function, taste, saliva, mood, and anxiety. PTGI was completed after 1, 6, and 12 months postoperatively; and it consisted of 21 questions with six possible answers to evaluate the five subscales [‘relating to others’ *(RO)*, ‘new possibilities’ *(NP)*, ‘personal strength’ *(PS)*, ‘appreciation of life’ *(AOL)*, and ‘spiritual change’ *(SC)*]. Of the 30 patients with OSCC who initially agreed to participate in the trial, 15 completed all questionnaires (n=15) over the one-year study period and were included in the final statistical evaluation (attrition rate, n=15).

#### Statistical evaluation

All HRQOL and PTG subscales were analyzed using a Friedman test. Significant test scores were further examined with a pairwise comparison using the Bonferroni-Dunn post hoc test. To investigate all possible influencing factors (e.g., sex, age, religious denomination, and marital status) on PTG, further correlation tests were performed. Mann-Whitney U tests for independent samples, and Wilcoxon tests for paired samples, were used to compare two groups. In addition, a descriptive statistical evaluation of pre- and postoperative HRQOL, as well as presentation of the different influencing variables on HRQOL, was performed. A Spearman's correlation analysis was performed to describe the relationship of HRQOL and PTG. All analyses were performed using SAS 9.4-(SAS Institute, Inc), Statistica 10.0 (Hamburg, Germany) and SPSS 24 (IBM; SPSS, Inc.) software. All tests resulting in a P-value <0.05 were considered statistically significant.

## Results

### 

#### Health-related quality of life (HRQOL)

**Figure 1:**
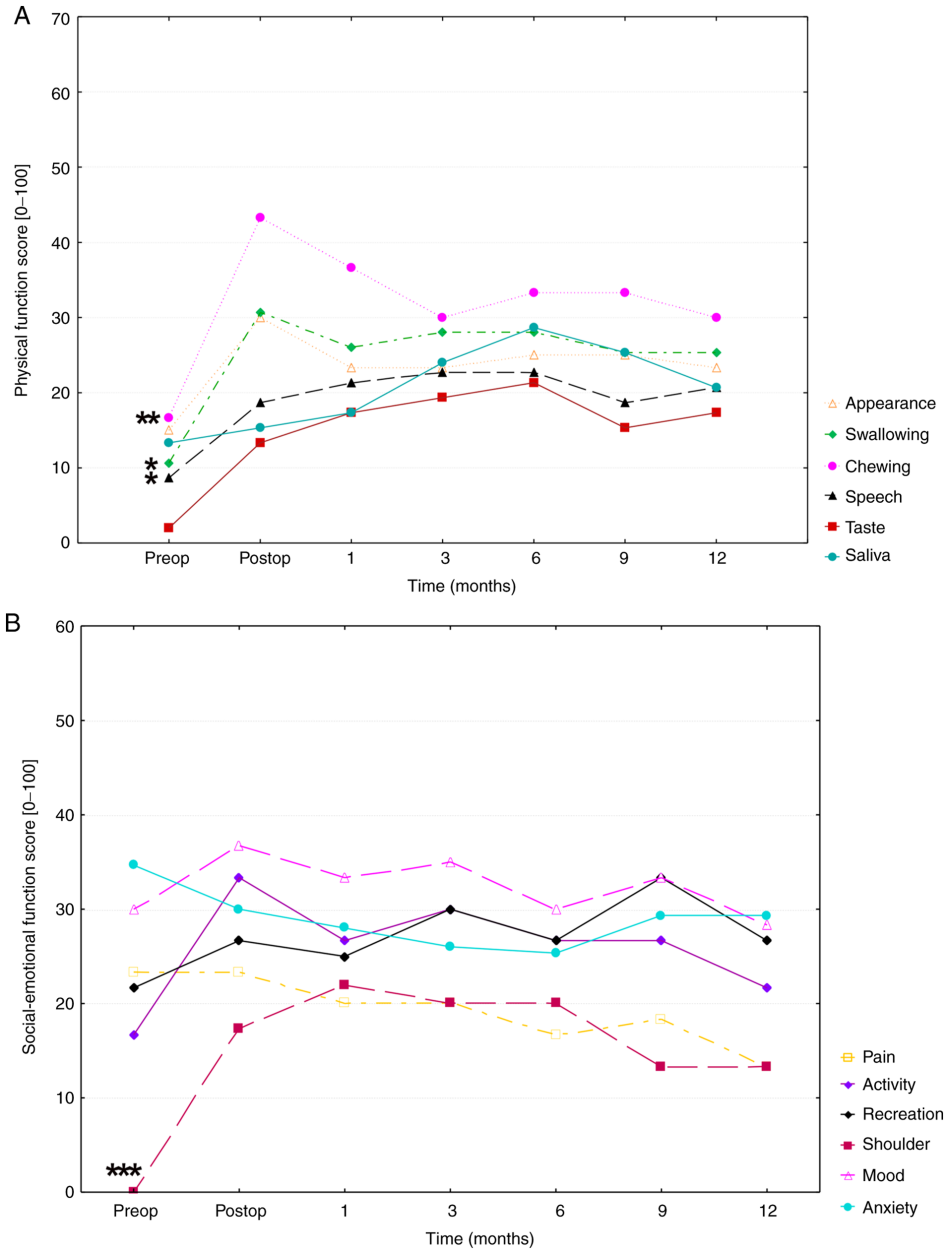
(A) Physical function score over time of the UW-QOL v4 questionnaire. The time of the mean scores of appearance, swallowing, chewing, speech, taste and saliva (physical subscales of the UW-QOL v4 questionnaire) are presented over a period from pre-surgery to 12 months post-surgery. High scores in the figure indicate poor HRQOL, whereas low scores indicate good HRQOL. (B) Social-emotional function score over time of the mean scores of pain, activity, shoulder, mood, anxiety and recreation (social-emotional subscales of the UW-QOL v4 questionnaire) over a period from pre-surgery to 12 months post-surgery. A Friedman test with Bonferroni-Dunn post hoc analysis was performed to compare differences between preoperative and mean postoperative physical and social-emotional function score values, respectively. Significances are indicated as asterisks (^*^P<0.05, ^**^P<0.01 and ^***^P<0.001). HRQOL, health-related quality of life. UW-QOL, University of Washington Quality of Life Version 4.

HRQOL evaluation revealed that both physical and socio-emotional HRQOL decreased in a pre- to-postoperative manner in all patients (Fig. 1A and B).

The mean UW-QoL v4 value in the physical function increased from 11 points preoperatively to 25 points postoperatively, indicating a decrease in physical HRQOL. In the area of physical function score, swallowing (MV, 24.9±25.6 SD; P=0.025), chewing (MV, 31.926.9 SD; P=0.002) and speech (MV, 19.1±20.7 SD; P=0.016) were the main factors responsible for postoperative deterioration in HRQOL. The mean value of the socio-emotional function increased from the preoperative (21 points) to postoperative (28 points) stage. Socio-emotional HRQOL was strongly influenced by patients' shoulder function (MV, 15.1±23.0; P<0.0001), and mood (mean, 32.4±25.2) which showed high mean values over the entire study period and was only subject to a few fluctuations. This result, however, was not statistically significant (P=0.88).

Statistical analysis of the preoperative UW-QoL v4 data with the one-year postoperative data indicated a significant decrease in the physical (P=0.011; 11 points preop, 23 points after 1 year) but not in the socio-emotional HRQOL (P=0.972; 21 points preop, 22 points after 1 year) over the one-year study period.

Correlation analysis results between various clinical baseline characteristics and the physical and socio-emotional HRQOL revealed that sex had a nearly significant effect on HRQOL (P=0.058). In general, men indicated a poorer HRQOL than women. On average, single patients reported a slightly poorer overall HRQOL than married patients. However, this difference was not significant (P=0.113). Furthermore, no significant effect of denominational affiliation (P=0.908) and patient's age (P=0.261) on HRQOL could be observed.

On average, patients with tumors classified as T1/T2, free microvascular flap reconstruction, and no additional radiotherapy (RT) were characterized by the poorest HRQOL scores. Patients (T1/T2) who received local defect reconstruction reported a better HRQOL. In T1/T2 carcinomas, socio-emotional HRQOL was rated as worse than physical HRQOL, regardless of the type of therapy used. In patients without free microvascular flap reconstruction and RT, no major difference could be observed between physical and socio-emotional HRQOL. In general, patients with T3/T4 carcinomas rated their physical HRQOL worse than those of other groups.

#### Post-traumatic growth (PTG)

**Figure 2:**
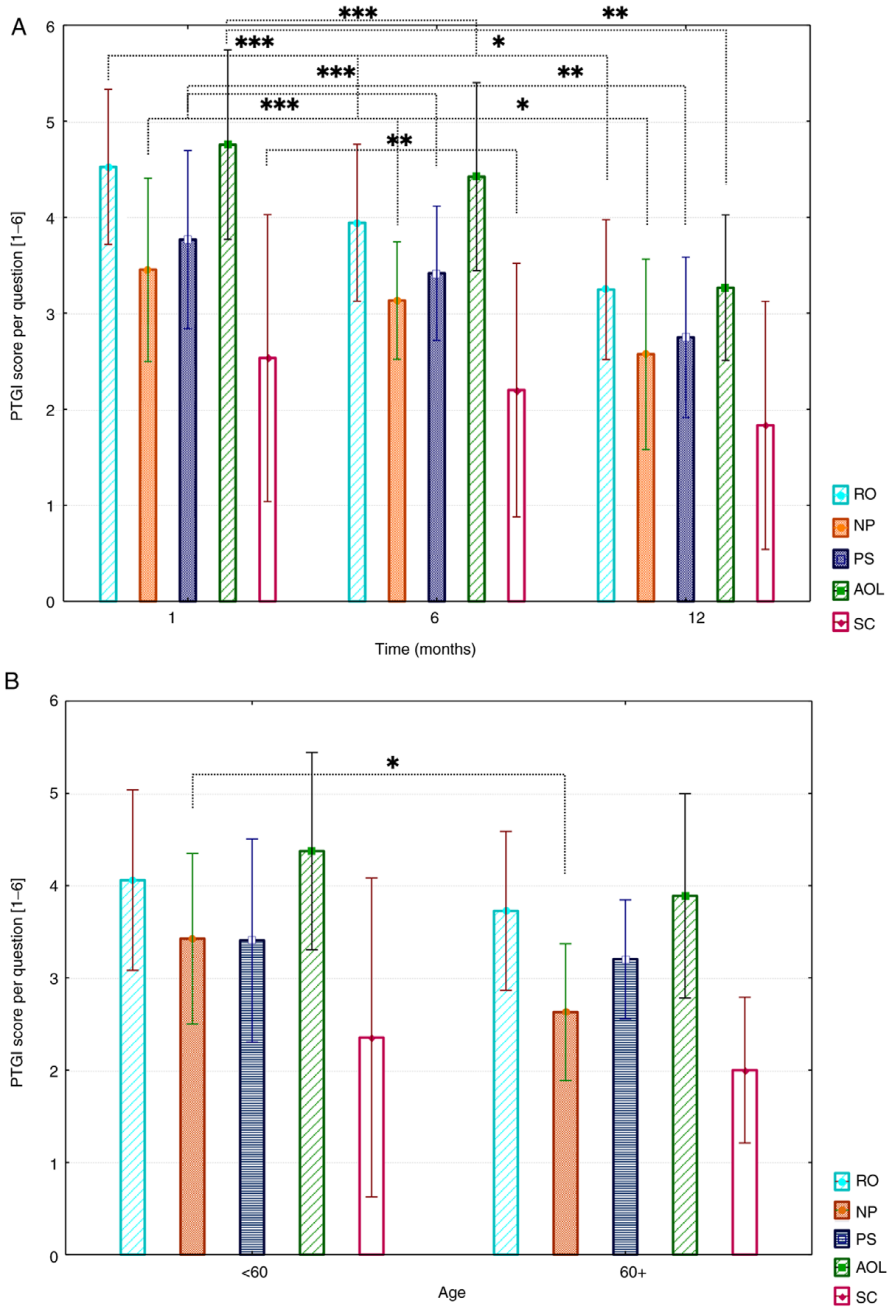
(A) Results of the PTGI questionnaire divided by subscales. MVs and SDs are demonstrated per question. A Friedman test with Bonferroni-Dunn post-hoc analysis was performed. ^*^P<0.05, ^**^P<0.01 and ^***^P<0.001. (B) Influence of age on overall PTG (1-, 6- and 12-months), MVs and SDs are presented. A Mann-Whitney U test was performed to determine statistical significance. ^*^P<0.05, ^**^P<0.01 and ^***^P<0.001 as indicated. PTGI, Posttraumatic Growth Inventory; MV, mean values; RO, relating to others; NP, new possibilities; PS, personal strength; AOL, appreciation of life; SC, spiritual change.

For PTG evaluation, three postoperative questionnaires (1, 6, and 12 months postoperative) were available for each patient. Mean PTG values per question (MV) for all patients are shown in Fig. 2A. The five subscales: ‘*Relating to others*’ (RO), ‘*new possibilities*’ (NP), ‘*personal strength*’ (PS), ‘*appreciation of life*’ (AOL), and ‘*spiritual change*’ (SC), composed of different number of questions, were separately evaluated. The mean values were used for comparison. The mean overall PTG score was 72.2±35.7 (95% CI, 69.9-74.5).

Results show that patients most strongly felt PTG shortly after surgery (1 month postoperatively). Mean values of all subscales revealed a PTG decrease over the study period, which was also observed uniformly for all subscales. The biggest changes were found in ‘*appreciation of life*’ (MV, 4.1±1.8; 95% CI, 3.6-4.6) and ‘*relating to others*’ (MV, 3.9±1.5; 95% CI, 3.5-4.3). ‘*Personal strength*’ (MV, 3.3±1.6; 95% CI, 2.8-3.8) and ‘*new possibilities*’ (MV, 3.0±1.6; 95% CI, 2.5-3.5) values slightly decreased in comparison. The lowest values were found in the *‘*spiritual change*’* subscale (MV, 2.2±2.7; 95% CI, 1.4-3.0). ‘*Appreciation of life*’ showed the highest decrease over all three timepoints (1 to 12 months postoperatively). Initially, a moderate decrease (MV, 4.8 to 4.4) from 1 to 6 months postoperatively was found, whereas a strong decrease from 6 months to 12 months postoperatively (MV 4.4 to MV 3.2) was observed. Additionally, for ‘*new possibilities*’ and ‘*personal strength*,’ such decrease was, however, found with initial lower values (NP MV 3.4 to MV 2.5; PS MV 3.8 to MV.8). ‘*Relating to others*’ showed a strong decrease from 1 month to 6 months postoperatively (RO MV 4.5 to MV 3.9). The global analysis showed a significant trend for all subscales (in particular: RO, P<0.001; NP, P<0.001; AOL, P<0.001; SC, P=0.001, and PS, P<0.001). Pairwise comparisons revealed significant differences between PTG subscales. For RO, between 1 month and 1 year (P<0.0001) and 6 months and 1 year (P=0.024). For NP, between 1 month and 1 year (P<0.0001) and 6 months and 1 year (P=0.019). For PS, between 1 month and 1 year (P<0.0001) and 6 months and 1 year (P=0.003). Also, significant differences could be observed between AOL subscale after 1 month and 1 year (P<0.0001) and 6 months and 1 year (P=0.002), and SC between 1 month and 1 year (P=0.006).

Analysis revealed slightly higher mean values in all five subscales for patients younger than 60 years of age (MV, 3.5) than those over 60 years (MV, 3.0). However, just for the ‘*new possibilities*’ subscale, the difference was statistically significant (P=0.040). A graphical representation is shown in Fig. 2B.

No significant correlation between PTG and sex or PTG and marital status has been found. The P-value from the ‘relating to others’ scale (P=0.04) indicates a significant correlation between the intensification of ‘relating to others’ and ‘spiritual change.’

#### Correlation between HRQOL and PTG

**Figure 3:**
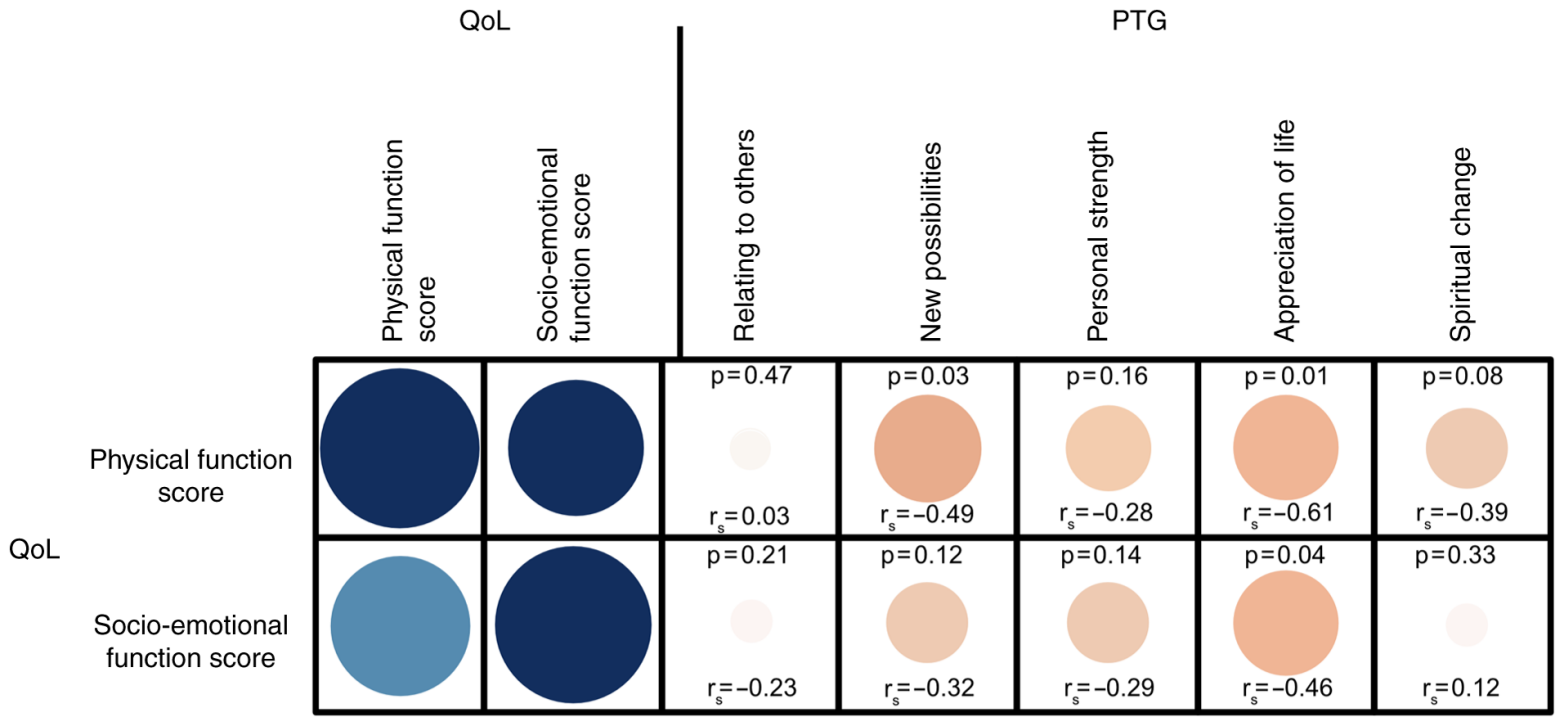
Correlogram *presenting* the correlation between HRQOL (social-emotional and physical function score) and all PTG subscales. The correlation strengths are illustrated in the form of size and color intensity. The correlogram is displayed with a color scale and shows a color gradient from blue (with scale 1), *to* white (with scale 0) to dark red (with scale-1). The size of the circle should additionally be an expression of the correlation strength. A Spearman's correlation analysis was performed to describe the relationship of HRQOL and PTG. P-value and Spearman's correlation coefficient (r_s_) are provided. HRQOL, health-related quality of life; QoL, quality of life; PTG, post-traumatic growth.

Statistical analyses results revealed a significant negative correlation between physical and socio-emotional HRQOL and the PTG subscales, ‘*new possibilities*’, and ‘*appreciation of life*’ (all P-values <0.05). All other correlation tests were not significant, as depicted in Fig. 3.

## Discussion

Head and neck cancers continue to be characterized by poor survival outcome and reduced HRQOL of patients (21,22). However, life-threatening diseases, such as cancer, are possible traumas that can trigger PTG (17). Many factors, such as hope, optimism, carer, and coping, may influence HRQOL and PTG. In this study, we characterize the relationship between influencing clinical factors, HRQOL, and PTG after extensive surgical OSCC treatment, and focus on data analyzable with available clinical standard questionnaires.

As already described in literature (23), the present analysis results showed a reduced physical- and socio-emotional HRQOL in all patients with OSCC. Comparable results, also achieved with the UW-QoL v4 questionnaire, were described one year postoperatively in patients with oropharyngeal carcinoma by Biazevic *et al* (24).

Chewing, swallowing, and speech were found to be the significant factors responsible for reduced postoperative physical HRQOL in this study. Most likely, these results are mainly due to the extensive intraoral OSCC resection and are comparable to the evaluation of Villaret *et al* (25).

The comparison of patients with tumors classified as T1/T2 showed a better HRQOL in those with local plastic defect reconstruction than in patients who received free microvascular flap reconstruction. In this context, it is important to note that there are important differences between the extents of tumor infiltration. Depending on the location, different surgical and reconstructive strategies are necessary. Interestingly, more than 50% of patients rated their health-related and overall HRQOL as ‘good’ or ‘satisfying,’ suggesting that despite the postoperatively reduced physical and socio-emotional function scores, the overall assessment was multifactorial influenced.

In this study, no sociodemographic factor, such as age, religion, and marital status, revealed a significant effect on HRQOL. This could be due to the small number of cases. Due to the burden of this dramatic life event, many patients who were preoperatively included in this trial showed poor postoperative compliance and were no longer willing to participate in the evaluation (drop-out rate, n=15). However, the particular value of the recent study lies in the complete dataset with no missing values over the entire study period. This is an important difference from many other studies where data are incomplete. Men rated their HRQOL worse than women, which, however, was on borderline significance (P=0.058). The influence of sex on postoperative HRQOL is debatable. While Rogers *et al* (26) reported a better HRQOL in women, other studies did not demonstrate a significant difference between the sexes or report contrarily (23,27).

Social support facilitates the coping process and reduces emotional discomfort. The ability to trust others and the feeling of support, understanding, and acceptance are considered important prerequisites for the emergence of PTG (19). The opportunity to express thoughts and fears gives patients the opportunity to complete mental processing (28,29).

In this study, all patients with OSCC reported a postoperative PTG. PTG was most strongly felt immediately after treatment (one month) and decreased over the one-year study period. The data in the literature on the development of PTG in the follow-up interval are not definite (30).

Thus far, the PTG experience is unclear as to whether it is real or just a self-calming defensive illusion in the course of mental processing (31-33). The question of the objectivity of the subjectively perceived PTG has already been a focus of research and is still being discussed controversially (34,35). In this context, the objectively collected PTGI values in this study should be noted to still indicate a positive perception even after one year. These results are consistent with those of the longitudinal study by Sharp *et al* (36). Additionally, the authors used the PTGI questionnaire by Tedeschi and Calhoun for PTG evaluation (17). ‘Appreciation of life’ had the highest mean value in this study, followed by the ‘relating to others’ and ‘personal strength’ subscales. In contrast to our results, however, ‘spiritual change’ occupied a stronger position among patients. The feeling of having ‘new possibilities’ was least felt by patients with head and neck cancers.

The findings of the study revealed slightly higher mean PTG values in all five subscales for younger patients than older patients. However, for the *‘new possibilities’* scale, the difference was statistically significant (P=0.040). Sharp *et al* (36) and others (37) reported similar results and assumed that older patients had a different perception due to earlier life experiences.

Although numerous studies have highlighted the effect of sociodemographic factors on PTG (30,37), no significant correlation between PTG, sex, marital status, or religious affiliation could be observed in this study. Despite the lack of significance, women tended to show higher values in all five PTG subscales, as also described by others (35,36,38,39). Although patients who identified themselves as non-denominational reported higher average values in three of the five subscales, spirituality did not show any significant effect on PTG in this study. In contrast, other studies reported that people with strong beliefs had less difficulty in coping with trauma (39,40).

A higher level of psychological distress is generally assumed to correlate with a higher level of PTG (41). We found a significant negative correlation between HRQOL and PTG in patients with OSCC. Patients reporting a poor HRQOL also reported poorer PTG values. This is corroborated by other studies (34,36,42). However, so far, no consistent relationship between HRQOL and PTG has been found (37).

After extensive tumor surgery, postoperative HRQOL and PTG are the most important factors for patients dealing with this traumatic life event. Many individually different factors can affect HRQOL and PTG, which can be assessed with available standardized questionnaires. Due to the suspicion of coherence in patients with OSCC, these should be used in clinical practice for postoperative combined screening to address individual patient needs and improve cancer follow-up care.

## Figures and Tables

**Table I:** Patient clinical characteristics.

Sex	Age	Profession	Religion	Marital status	Tumor localization	pT	pN	pM	Reconstruction
Male	48	Gardener	None	Not married	Anterior mouth floor	4	2	0	Distant flap
Female	48	Psychotherapist	Evangelic	Divorced	Lateral upper alveolar process	2	0	0	Local
Male	67	Baker	Catholic	Not married	Lateral mouth floor	1	0	0	Local
Male	47	Warehouse worker	Muslim	Not married	Cheek	1	0	0	Distant flap
Female	94	Secretary	Evangelic	Widowed	Lateral tongue	Cis	0	0	Local
Male	48	Educator	Catholic	Not married	Palate	1	0	0	Local
Male	67	Manager	Evangelic	Married	Anterior mouth floor	2	0	0	Distant flap
Male	63	Caregiver	Catholic	Married	Lateral lower alveolar process	4	0	0	Distant flap
Male	64	Caregiver	Catholic	Married	Lateral lower alveolar process	4	0	0	Distant flap
Female	46	Banker	None	Married	Lateral upper alveolar process	4	0	0	Distant flap
Male	56	Bricklayer	None	Not married	Lateral tongue	2	0	0	Local
Male	56	Gardener	None	Not married	Cheek	2	0	0	Distant flap
Male	54	Machinist	Evangelic	Married	Anterior mouth floor	1	0	0	Distant flap
Female	67	Secretary	Catholic	Married	Lower lip	1	0	0	Local
Female	76	Artist	None	Married	Lateral tongue	1	0	0	Local

## Data Availability

The datasets used and/or analyzed during the current study are available from the corresponding author on reasonable request.
